# Supplementary material for: Genesis of Hawaiian lavas by crystallization of picritic magma in the deep mantle
Source: Nat Commun. 2023 Mar 13;14:1382. doi: 10.1038/s41467-023-37072-0 (PMC10011491; doi:10.1038/s41467-023-37072-0)
Supplement: Supplementary file 1 — Supplementary Information [file 41467_2023_37072_MOESM1_ESM.pdf]

Supplementary Information

Genesis of Hawaiian lavas by crystallization of picritic magma in the deep mantle

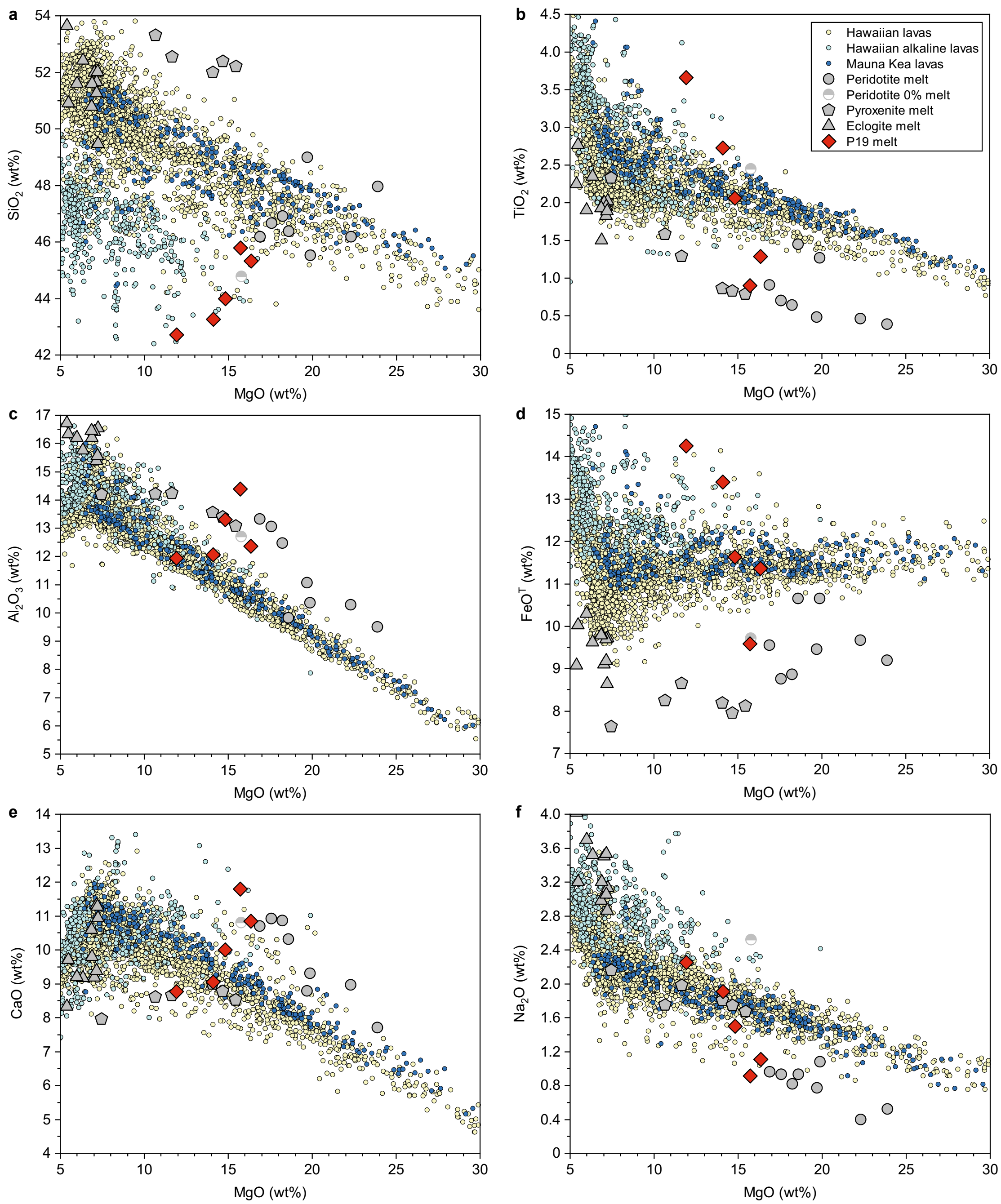

Supplementary Figure 1. Partial melt compositions of possible source materials of Hawaiian lavas (a to f), showing that Hawaiian lavas are too high in FeO^T to be consistent with previous models. The data of Hawaiian lavas are from the GEOROC database. Mauna Kea lavas of Hawaii are from ref. [1] for comparison, showing the similar composition trend with Hawaiian lavas. P19 melt, partial melts of P19 at 3.0 GPa (this

study). Peridotite melt, partial melts of mantle peridotite at 3.0-4.0 GPa (ref. [2]). Peridotite 0% melt, near 0% melts of mantle peridotite at 3.0 GPa (ref. [3]). Pyroxenite melt, partial melts of olivine-free pyroxenite[4]. Eclogite melt, partial melts of eclogite at 3.0-5.0 GPa (refs. [5,6,7,8]).

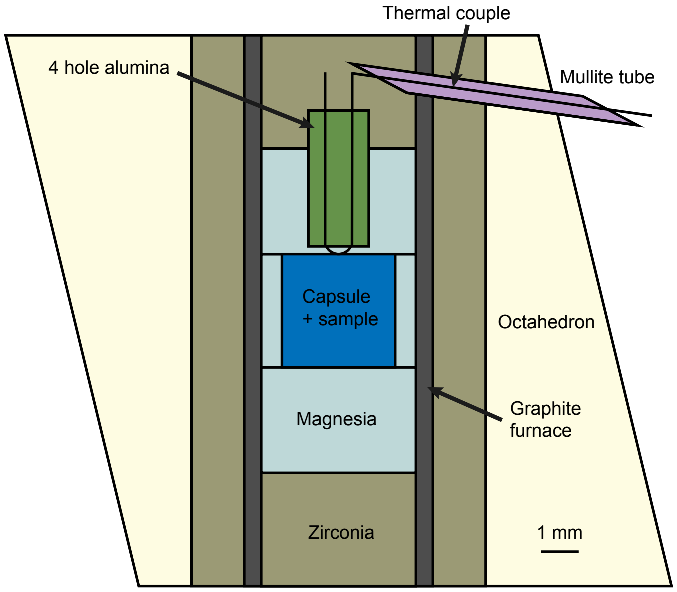

Supplementary Figure 2. Schematic cross section of the 18/12 cell assembly.

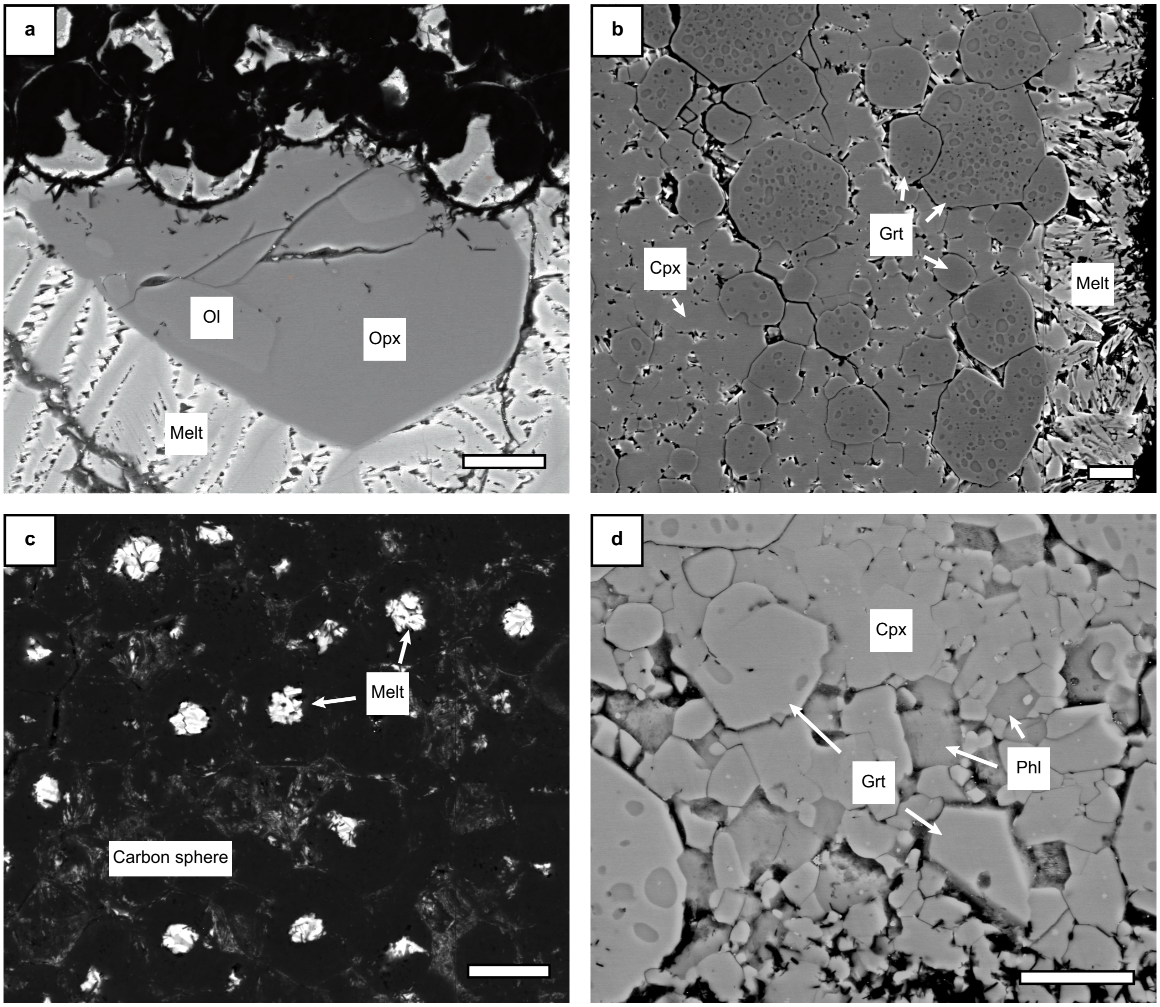

Supplementary Figure 3. Backscattered electron images of experimental run products. **a**, R1238, 1,400 °C; **b**, R1243, 1,300 °C; **c**, R1239, 1,250 °C containing melt traps in the carbon spheres; **d**, R1246, 1,200 °C. Scale bars, 20 μm. Ol, olivine; Opx, orthopyroxene; Cpx, clinopyroxene; Grt, garnet; Phl, phlogopite.

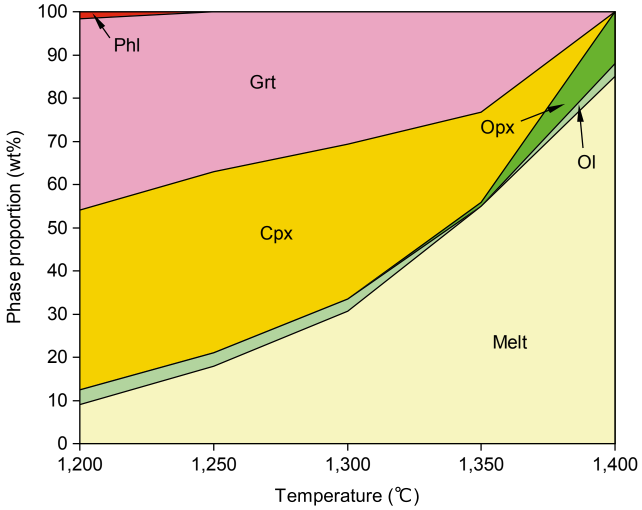

Supplementary Figure 4. Calculated phase proportions of P19 at 3.0 GPa with different temperatures. The solid lines are the connection between the data points. Each data point represents corresponding temperature which is not shown for simple and clear. Mineral abbreviations are given in Supplementary Figure 3.

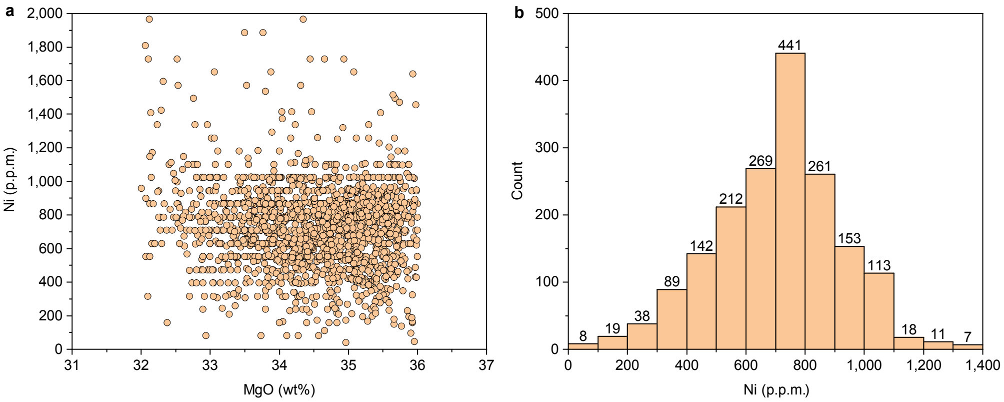

Supplementary Figure 5. Ni concentrations in harzburgite opx. a, Ni plotted against MgO; **b,** Histograms of Ni concentrations. Data are taken from the GEOROC database. The Ni concentrations of harzburgite orthopyroxene is 722 p.p.m. in mean, and the most frequent counts are 700-800 p.p.m..

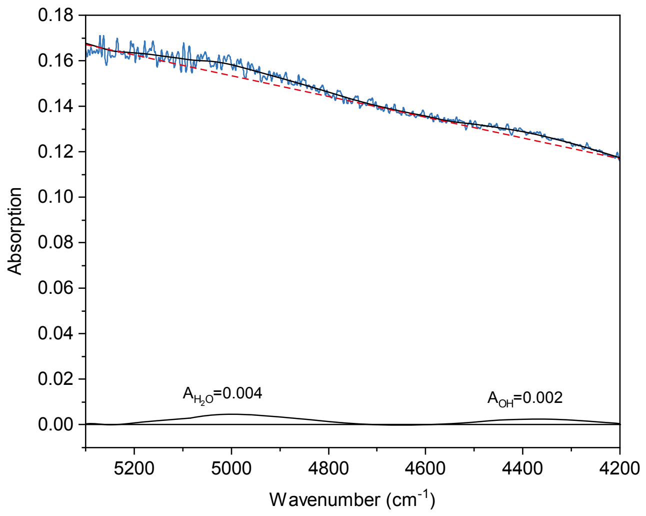

Supplementary Figure 6. Infrared spectrum of the starting glass material P19 showing typical absorption of total water (H_2O molecules and OH groups). The top image is the raw spectrum (blue solid line) and smoothed spectrum (black solid line) showing the straight baseline (red dash line) background fit between 5300 and 4200 cm^{-1}. The bottom image is the background corrected spectrum showing the corresponding range of baseline. Lambert-Beer parameters used to quantify total water concentration are as follows: thickness (d) = 0.0152 cm, density (ρ) = 3120 g l^{-1} for picritic glass from ref. [9], molar absorption coefficient (ε_{H2O}) = 0.71 l mol^{-1} cm^{-1} and (ε_{OH}) = 0.66 l mol^{-1} cm^{-1} from ref. [10] and absorbance (A_{H2O}) = 0.004 and (A_{OH}) = 0.002 from the peak height of the bottom image. This yields a total water concentration of 0.33 wt%.

Supplementary Table 1. Experimental conditions and compositions of run products

Phase	N*	SiO_2	TiO_2	Al_2O_3	FeO^T	MnO	MgO	CaO	Na_2O	K_2O	P_2O_5	Cr_2O_3	Total	Mg#	Mode†
Starting material															
P19	6	46.5 (1)	0.80 (3)	13.13 (8)	9.09 (9)	0.10 (5)	18.5 (1)	10.33 (7)	0.81 (3)	0.48 (2)	0.10 (2)	0.19 (5)	100.00	0.78	100
R1246, 3GPa, 1200°C, 21h, R2‡=0.03, Kd§=0.31															
Melt	3	42.7 (13)	3.7 (2)	11.9 (8)	14.3 (17)	0.10 (3)	11.9 (4)	8.8 (16)	2.3 (4)	3.6 (3)	0.8 (2)	0.02 (3)	100.00	0.60	9.1
Ol	3	39.5 (3)	0.04 (1)	0.04 (1)	16.5 (1)	0.05 (3)	44.2 (2)	0.24 (2)	0.02 (1)	-	0.03(2)	-	100.62	0.83	3.3
Cpx	3	53.4 (3)	0.41 (3)	4.2 (3)	5.9 (2)	0.06 (3)	17.95 (9)	16.7 (1)	1.06 (4)	0.02 (1)	-	0.09 (5)	99.81	0.84	41.7
Grt	3	41.6 (3)	0.55 (4)	22.8 (2)	10.7 (3)	0.16 (4)	18.2 (2)	5.8 (1)	0.03 (2)	-	0.03 (1)	0.35 (3)	100.17	0.75	44.3
Phl	3	38.7 (5)	2.97 (8)	15.5 (2)	6.6 (1)	0.02 (1)	21.2 (3)	0.2 (2)	0.25 (6)	9.9 (4)	-	0.15 (2)	95.49	0.85	1.6
R1239, 3GPa, 1250°C, 24h, R2=0.40, Kd=0.38															
Melt	7	43.3 (5)	2.7 (3)	12.1 (10)	13.4 (5)	0.08 (6)	14.1 (5)	9.1 (5)	1.9 (1)	3.1 (3)	0.32 (9)	0.03 (2)	100.00	0.65	18
Ol	4	39.1 (2)	0.05 (3)	0.11 (4)	15.7 (2)	0.08 (3)	43.9 (5)	0.28 (4)	0.007 (6)	-	0.02 (1)	-	99.31	0.83	3
Cpx	3	52.4 (2)	0.34 (1)	4.8 (4)	6.1 (4)	0.07 (2)	19.0 (2)	15.5 (6)	0.90 (4)	0.02 (1)	0.04 (1)	0.15 (6)	99.27	0.85	42
Grt	3	41.7 (4)	0.52 (2)	22.9 (2)	9.8 (3)	0.08 (1)	19.1 (3)	6.0 (2)	0.02 (1)	-	0.04 (3)	0.33 (4)	100.45	0.78	37
R1243, 3GPa, 1300°C, 24h, R2=0.11, Kd=0.41															
Melt	11	44.0 (6)	2.1 (2)	13.3 (6)	11.6 (10)	0.08 (3)	14.8 (8)	10.0 (6)	1.5 (4)	2.3 (2)	0.22 (4)	0.06 (4)	100.00	0.69	30.6
Ol	4	40.4 (4)	0.04 (2)	0.10 (1)	14.6 (5)	0.11 (3)	45.5 (1)	0.24 (3)	0.01 (1)	-	0.03 (2)	0.02 (1)	101.07	0.85	2.9
Cpx	3	52.5 (2)	0.32 (5)	5.4 (3)	6.2 (1)	0.05 (4)	19.1 (5)	15.3 (3)	0.68 (2)	0.013 (6)	0.03 (1)	0.15 (4)	99.80	0.85	35.9
Grt	3	42.3 (2)	0.45 (5)	23.4 (2)	9.1 (4)	0.17 (2)	19.1 (5)	6.21 (7)	0.02 (1)	-	0.04 (2)	0.38 (1)	101.18	0.79	30.6
R1244, 3GPa, 1350°C, 24h, R2=0.23															
Melt	8	45.3 (8)	1.29 (7)	12.4 (5)	11.4 (8)	0.08 (4)	16.4 (5)	10.8 (4)	1.1 (2)	1.1 (3)	0.12 (5)	0.04 (3)	100.00	0.72	55
Opx	3	54.5 (7)	0.07 (2)	4.2 (10)	6.9 (2)	0.10 (3)	30.6 (3)	2.7 (1)	0.12 (4)	-	-	0.22 (7)	99.41	0.89	0.8
Cpx	3	53.4 (4)	0.12 (2)	4.5 (4)	5.6 (4)	0.05 (2)	22.5 (9)	12.7 (14)	0.48 (5)	0.007 (6)	0.03 (1)	0.15 (4)	99.48	0.88	21
Grt	3	41.8 (1)	0.32 (4)	23.1 (1)	7.1 (7)	0.14 (3)	20.2 (3)	5.9 (2)	0.01 (1)	-	0.03 (1)	0.5 (1)	99.03	0.84	23.2
R1238, 3GPa, 1400°C, 24h, R2=0.02, Kd=0.33															
Melt	7	45.8 (3)	0.90 (4)	14.4 (2)	9.6 (2)	0.12 (2)	15.7 (2)	11.8 (2)	0.91 (7)	0.54 (6)	0.12 (2)	0.15 (4)	100.00	0.74	85
Ol	3	40.5 (1)	0.02 (1)	0.17 (1)	9.7 (2)	0.08 (2)	48.51 (2)	0.34 (3)	-	-	0.01 (1)	0.08 (4)	99.42	0.90	3
Opx	3	53.09 (4)	0.10 (2)	6.8 (5)	5.8 (2)	0.09 (2)	30.2 (4)	2.5 (1)	0.06 (2)	-	-	0.59 (8)	99.30	0.90	12
Average melt															
AM-40‖		44.2 (7)	2.13 (15)	12.8 (6)	12.0 (8)	0.09 (4)	14.6 (5)	10.1 (7)	1.5 (3)	2.1 (2)	0.31 (8)	0.06 (3)	100.00	0.68	40
AM-28¶		43.8 (8)	2.44 (17)	12.4 (7)	12.7 (10)	0.08 (4)	14.3 (5)	9.7 (8)	1.7 (3)	2.5 (3)	0.36 (9)	0.04 (3)	100.00	0.67	28

*N, the number of electron probe analysis used to obtain the average compositions.

Mg# is the mole ratio calculated as Mg/(Mg + Fe); total Fe is given as FeO (FeO^T).

†Mode, phase proportions (in wt%) calculated by least-squares mass balance.

‡R2, sum of squared residuals in mass balance calculations.

§Kd, the exchange coefficient of Fe and Mg between olivine and melt.

‖AM-40, the average composition of P19 melt with MF ranging from 85% to 9%.

¶AM-28, the average composition of P19 melt with MF ranging from 59% to 9%.

The values in parentheses are one sigma standard deviation with respect to mean based on replicate electron microprobe analyses and are reported as least units cited; 13.5 (12)

should be read as 13.5 +/- 1.2 wt%.

Supplementary Table 2. Calculated Ni concentrations in P19 and its partial melts

Run. no	30.14*	R1238	R1244	R1243	R1239	R1246	AM-40	AM-28
T (°C)	1,540	1,400	1,350	1,300	1,250	1,200		
D ol-melt	4.12	5.72		6.76	7.54	9.91		
D opx-melt	1.69	1.94		2.04	2.14	2.51		
D cpx-melt	1.01	1.07		1.05	1.06	1.20		
D grt-melt	0.41	0.38		0.32	0.30	0.32		
bulk D	3.31	2.69	0.98[‡]	0.97	0.96	1.07		
Ni in melt[†]	728	581	735	744	756	687	700	730

[*]We used the phase compositions and proportions of Run 30.14 (ref. [2]) to calculated the Ni concentrations in P19 due to their similar compositions.

[†]Calculated Ni concentrations (p.p.m.) in melt using the same method in ref. [4] based on 2,000 p.p.m. Ni in mantle peridotite.

D mineral-melt is the calculated partition coefficient of Ni between crystals and melt.

Bulk D is the calculated bulk partition coefficient of Ni as described in ref. [11].

[‡]Bulk D of R1244 is estimated according to R1243 and R1239, because no olivine was observed in run product.

Supplementary Table 3. Modeling of reaction between P19 melt and harzburgite orthopyroxene

Phase	SiO_2	TiO_2	Al_2O_3	FeO^T	MnO	MgO	CaO	Na_2O	K_2O	P_2O_5	Cr_2O_3	Total	Ni	Initial olivines*	
														Fo	NiO
Opx†	56.92	0.05	1.90	5.58	0.13	34.47	0.64	0.07	0.01	0.01	0.43	100.21	722		
AM-40	44.2	2.13	12.8	12.0	0.09	14.6	10.1	1.5	2.1	0.31	0.06	99.89	700	88.7	0.67
AM-28	43.8	2.44	12.4	12.7	0.08	14.3	9.7	1.7	2.5	0.36	0.04	100.02	730	88.0	0.70
AM-40 + Opx															
10%‡	45.49	1.92	11.72	11.40	0.10	16.57	9.14	1.39	1.91	0.28	0.10	100.02	702	90.4	0.60
20%	46.76	1.71	10.63	10.75	0.10	18.56	8.20	1.24	1.70	0.25	0.14	100.04	704	91.9	0.54
30%	48.03	1.50	9.54	10.10	0.10	20.55	7.25	1.10	1.49	0.22	0.17	100.05	707	93.1	0.49
AM-28 + Opx															
10%	45.14	2.20	11.36	11.95	0.09	16.32	8.76	1.53	2.27	0.33	0.08	100.03	729	89.9	0.63
20%	46.45	1.96	10.31	11.24	0.09	18.33	7.86	1.37	2.02	0.29	0.12	100.04	728	91.5	0.56
30%	47.76	1.72	9.26	10.53	0.10	20.35	6.96	1.20	1.77	0.25	0.16	100.06	728	92.8	0.51

*The calculated composition of the initial olivines crystallized from the corresponding melts.

†Opx, average composition of harzburgite orthopyroxene (Supplementary Figure 5).

‡Proportions of orthopyroxene.

The Ni concentrations in AM-40 and AM-28 are from Supplementary Table 2.

Supplementary Table 4. The rare earth element compositions of harzburgite orthopyroxene and estimated parental melts

	Opx*	AM-40 + 30% Opx	AM-28 + 30% Opx
La	0.0108	10.209	12.037
Ce	0.0354	9.897	11.627
Nd	0.0495	8.207	9.499
Sm	0.0288	6.239	7.023
Gd	0.0539	4.917	5.394
Dy	0.0558	2.849	2.798
Er	0.0670	2.596	2.484
Yb	0.1119	2.118	1.886
Lu	0.0276	2.082	1.853

*Opx, average composition of harzburgite orthopyroxene. Data are taken from the GEOROC database.

Supplementary References

1. Rhodes, J. M. & Vollinger, M. J. Composition of basaltic lavas sampled by phase-2 of the Hawaii Scientific Drilling Project: Geochemical stratigraphy and magma types. *Geochem. Geophys. Geosyst.* **5**, Q03G13 (2004).
2. Walter, M. J. Melting of Garnet Peridotite and the Origin of Komatiite and Depleted Lithosphere. *J. Petrol.* **39**, 29-60 (1998).
3. Davis, F. A., Hirschmann, M. M. & Humayun, M. The composition of the incipient partial melt of garnet peridotite at 3GPa and the origin of OIB. *Earth Planet. Sci. Lett.* **308**, 380-390 (2011).
4. Sobolev, A. et al. The Amount of Recycled Crust in Sources of Mantle-Derived Melts. *Science* **316**, 412-417 (2007).
5. Yasuda, A., Fujii, T. & Kurita, K. Melting phase relations of an anhydrous mid-ocean ridge basalt from 3 to 20 GPa: Implications for the behavior of subducted oceanic crust in the mantle. *J. Geophys. Res.* **99**, 9401-9414 (1994).
6. Yaxley, G. & Green, D. Reactions between eclogite and peridotite: Mantle refertilisation by subduction of oceanic crust. *Schweiz. Mineral. Petrogr. Mitt.* **78**, 243-255 (1998).
7. Pertermann, M. & Hirschmann, M. M. Partial melting experiments on a MORB-like pyroxenite between 2 and 3 GPa: Constraints on the presence of pyroxenite in basalt source regions from solidus location and melting rate. *J. Geophys. Res.* **108**, 2125 (2003).
8. Spandler, C., Yaxley, G., Green, D. H. & Rosenthal, A. Phase Relations and Melting of Anhydrous K-bearing Eclogite from 1200 to 1600°C and 3 to 5 GPa. *J. Petrol.*

49, 771-795 (2008).

9. Ohtani, E. & Maeda, M. Density of basaltic melt at high pressure and stability of the melt at the base of the lower mantle. *Earth Planet. Sci. Lett.* **193**, 69-75 (2001).

10. Ohlhorst, S., Behrens, H. & Holtz, F. Compositional dependence of molar absorptivities of near-infrared OH- and H_2O bands in rhyolitic to basaltic glasses. *Chem. Geol.* **174**, 5-20 (2001).

11. Sobolev, A. V., Hofmann, A. W., Sobolev, S. V. & Nikogosian, I. K. An olivine-free mantle source of Hawaiian shield basalts. *Nature* **434**, 590-597 (2005).
